# Perinatale Palliativbetreuung im zeitlichen Wandel: eine longitudinale Beobachtungsstudie

**DOI:** 10.1055/a-2685-1273

**Published:** 2025-09-24

**Authors:** Carmen Edda Jakubowicz, Andreas Walter Flemmer, Esther Sabine Schouten

**Affiliations:** 1 Neonatologie der Kinderklinik am Perinatalzentrum der LMU München, Campus Großhadern, Kinderklinik und Kinderpoliklinik im Dr. von Haunerschen Kinderspital am Klinikum der Universität, München, Germany

**Keywords:** Palliativmedizin, Grenze der Lebensfähigkeit, Zurückhalten lebensverlängernder Therapie, palliative care, withholding life sustaining therapy, border of viability

## Abstract

Neonatale Todesfälle treten großteils auf, nachdem eine Entscheidung, lebensunterstützende Maßnahmen zu beenden, getroffen wurde [1–7]. Neugeborene sterben selten unerwartet sondern überwiegend nach einer Entscheidung zur Therapiezieländerung. Der Entscheidungsweg dazu hat sich über die Jahre deutlich gewandelt und die praktische Umsetzung variiert stark zwischen den einzelnen Neugeborenenintensivstationen [1, 8]. Ziel der Studie war es, die näheren Umstände neonataler Todesfälle einer universitären Neonatologie in einem definierten Zeitraum zu evaluieren und Veränderungen im Laufe der Zeit festzuhalten. Während des 10-jährigen Beobachtungszeitraumes wurden am LMU Klinikum München 41 543 Kinder entbunden, von diesen sind 348 Kinder peri- oder postnatal verstorben. Im Kreißsaal verstarben 248 Kinder. Auf der Neugeborenenintensivstation verstarben von den 10 908 im Beobachtungszeitraum versorgten Kindern insgesamt 97. Ein zunehmend proaktiverer Ansatz hat in den letzten Jahren dazu geführt, dass dieprimäre Palliativversorgung Extremfrühgeborener häufiger abgelöst wurde durch einen Therapieversuch an der Grenze der Lebensfähigkeit. Da keine Änderung der Leitlinie zum Vorgehen bei Frühgeburtlichkeit an der Grenze der Lebensfähigkeit während des Datenerhebungszeitraumes vorgenommen wurde, ist diese Veränderung eher dem Zusammenwirken elterlicher Wunsch- und Erwartungshaltung und ärztlichem Handeln zuzuschreiben.

## Abstract (en)

Advances in perinatal medicine have contributed to significantly improved survival of newborns. While some infants die despite extensive medical treatment, a larger proportion dies after a decision to withdraw life-sustaining therapy is made. The approaches to these decisions have significantly changed over the years, and their practical implementation still varies greatly between different neonatal intensive care units. The aim of this study was to evaluate the circumstances surrounding all neonatal deaths in a university neonatal setting in Germany over a ten-year period and to document changes over time. During the 10-year study period, 41,543 children were born at the LMU university hospital Munich, while 348 children died during this time. Of these, 248 children passed away in the delivery room. A total of 10,908 children received medical care in the neonatal wards (two level III NICUs and two intermediate care units). Of these, more than half (56%) were term infants, and only about 1% wereat the border of viability. On the neonatal intensive care unit, a total of 97 newborns died. A more proactive approach has led to primary palliative care for extremely preterm infants being increasingly replaced by attempts at therapy at the threshold of viability. Since there was no change in the guidelines for the management of preterm infants at the border of viability during the data collection period, this shift could be attributed more likely to the interaction of parental preferences and expectations and medical decision-making.

## Einleitung

Nach Angaben des statistischen Bundesamtes versterben in Deutschland jährlich ca. 4000 Kinder und Jugendliche bis zum 18. Lebensjahr. Ungefähr 60% dieser Kinder finden den Tod im 1. Lebensjahr und zwei Drittel davon in der Neonatalperiode 9 .

Während die Zahlen für allgemeine Kindersterblichkeit über die letzten Jahrzehnte kontinuierlich zurückgingen und laut WHO zwischen 1990 und 2013 um 56% fielen, ist die Mortalität für Neugeborene zuletzt langsamer gesunken. Zwar verringerte sie sich zwischen 1990 und 2013 um etwa 40%, dennoch sind neonatale Todesfälle im Kreißsaal und auf der Neugeborenenintensivstation beständig ein Teil der klinischen Realität 10 11 . In der Neonatologie kam es gegen Ende des 20. Jahrhunderts zu stark veränderten Überlebenszahlen, insbesondere durch das Überleben sehr unreifer Frühgeborener und schwer erkrankter Neugeborener. Nicht nur die Mortalität änderte sich, auch das Spektrum der Erkrankungen, welche zum Tod Neugeborener führten, unterlag einem Wandel 12 . Diese Veränderungen sind neben dem technischen Fortschritt in der Therapie auch durch veränderte pränatale Prophylaxen und erhöhte Schwangerschaftsabbruchraten bei verbesserter Früherkennung von schwerwiegenden Fehlbildungen erklärbar 13 . Im Jahr 1973 wurde erstmals öffentlich diskutiert, lebensunterstützende Maßnahmen bei Neugeborenen mit unheilbaren Erkrankungen oder Fehlbildungen, welche mit einer infausten Prognose und/oder enorm eingeschränkter Lebensqualität assoziiert waren, aktiv und bewusst zu beenden 14 . Schrittweise fanden in den Jahren danach die Praktiken, intensivmedizinische Maßnahmen bei kranken Neugeborenen und Frühgeborenen primär zurückzuhalten oder zu beenden den Weg in den Aufgabenbereich und das Bewusstsein der Neonatologie 4 15 16 . Die Entscheidungsfindung zur Therapiezieländerung bei Neugeborenen hat sich über die Jahre deutlich verändert und neben dem theoretischen Hintergrund variiert auch die praktische Umsetzung stark zwischen den einzelnen Neugeborenenintensivstationen innerhalb Deutschlands, aber auch international 1 8 . Mehrere Studien zeigen, dass neonatale Todesfälle in unterschiedlichem, aber insgesamt zunehmendem Maß – in 25–93% – nach einer Entscheidung, lebensunterstützende Maßnahmen zu beenden eintreten 1 2 3 4 5 6 7 . Heutzutage sterben Neugeborene also selten unerwartet sondern überwiegend nach einer bewussten Entscheidung zur Therapiezieländerung.

Diese Unterschiede in der praktischen Umsetzung werden auch durch einen uneinheitlichen Gebrauch von Definitionen verursacht, Begrifflichkeiten und Kategorien für Maßnahmen und Patientengruppen. Die wissenschaftliche Evidenz für angewandte Methoden bei perinatalen Palliativbetreuungen ist überschaubar, und bisher erhoben nur wenige Studien detailliertere Daten zu den konkreten Umständen neonataler Todesfälle. Sowohl national als auch international wurde beispielsweise nur selten zwischen verstorbenen Neugeborenen im Kreißsaal und auf der Neugeborenenintensivstation differenziert und Studien, die neonatale Todesfälle im Kreißsaal analysieren, behandelen nahezu ausschließlich die Problematik der Betreuung Extremfrühgeborener an der Grenze der Lebensfähigkeit 2 17 18 19 .

Neben der Tatsache, dass der Tod eines Neugeborenen ein einschneidendes Ereignis für Eltern und eine Herausforderung für das medizinische Behandlungsteam bedeutet, machen nationale und internationale Unterschiede sowie die begrenzte Datenlage eine sorgfältige und vollständige Exploration perinatale und neonataler Palliativsituationen zu einer Angelegenheit von wissenschaftlichem Interesse. Eine standardisierte Erfassung von vordefinierten Patientengruppen und Maßnahmen in der Palliativversorgung würde zudem einen internationalen Vergleich ermöglichen.

Ziel der vorgelegten Studie ist es, die näheren Umstände aller neonatalen Todesfälle einer universitären Neonatologie in Deutschland in einem definierten Zeitraum zu evaluieren und Veränderungen über die Zeit zu analysieren.

## Methodik

In der Neonatologie des LMU Klinikums München werden pro Jahr ungefähr 4100 Kinder geboren. Mit in umliegenden Krankenhäusern geborenen und zuverlegten kranken Neugeborenen werden pro Jahr über 1000 Kinder stationär intensivmedizinisch betreut. I

In den Kreißsälen und auf den Neugeborenenintensivstationen (NICU) kommt es dabei jährlich zu etwa 35 neonatalen Todesfällen und Totgeburten.

Ziel der Studie war es, die näheren Umstände aller perinatalen Todesfälle in einem definierten Zeitraum von 10 Jahren (01.01.2006 bis zum 31.12.2015) zu evaluieren.

Für die Datenerhebung wurden alle Lebendgeborenen sowie alle Totgeburten ab einem Gestationsalter von 22 0/7 Schwangerschaftswochen eingeschlossen. Ausgeschlossen wurden alle Neugeborenen, die mit einem Gestationsalter unter 22 0/7 Schwangerschaftswochen tot auf die Welt kamen oder verstarben. Der Graubereich an der Grenze der Lebensfähigkeit wurde definiert als 22 0/7–23 6/7 SSW entsprechend der jeweiligen im Erhebungszeitraum gültige Leitlinie der Arbeitsgemeinschaft der Wissenschaftlichen Medizinischen Fachgesellschaften (AWMF).

## Datenerhebung und Ethik

Die Datenerhebung erfolgte in Form einer retrospektiven Datenextraktion nach Aktenlage.

Das Studienprotokoll wurde von der Ethikkommission der Medizinischen Fakultät der LMU München begutachtet und positiv bewertet (Aktenzeichen 17–073). Die Studie wurde in Übereinstimmung mit der aktuellsten Version der Deklaration von Helsinki durchgeführt 20 .

Bereits bei der Datenerhebung wurden die Daten pseudonymisiert und in pseudonymisierter Form weiterbearbeitet und ausgewertet. Die Daten wurden gemäß Artikel 27 Datenschutz Abschnitt 4 des Bayerischen Krankenhausgesetzes erhoben, verarbeitet und aufbewahrt.

## Datenauswertung

Da die Voraussetzungen zum Zeitpunkt des Versterbens stark variieren, wurden die verstorbenen Früh- und Neugeborenen unterschiedlichen Kategorien zugeteilt, um die genaueren Umstände des Sterbens im Rahmen einer Therapiezieländerung interpretieren und vergleichen zu können. So ist eine primär palliative Betreuung eines extrem Frühgeborenen mit 22+0/7 Schwangerschaftswochen bei unaufhaltsamen Wehen der Mutter im Rahmen eines schweren Amnioninfektionssyndromes nach einem ausführlichen Gespräch mit den Eltern anders zu kategorisieren, als beispielsweise eine völlig unerwartet frustrane Reanimation im Kreißsaal bei einem reifen Neugeborenen mit Asphyxie nach Plazentalösung. Auch die Palliativsituationen auf der NICU können sich sehr stark unterscheiden.

Damit zusätzlich die Möglichkeit einer guten internationalen Datenvergleichbarkeit gegeben ist, orientieren sich die hier verwendeten Sterbekategorien weitestgehend an bereits zuvor verwendeten und 2015 publizierten Einteilungen nach Koper et al. im Weiteren bezeichnet als *Mode of Death* (MOD), siehe Tab. 1 21 . Die Kategorisierung der einzelnen Todesfälle wurde ausschließlich durch die Erstautorin vorgenommen, um eine einheitliche Folgerichtigkeit zu gewährleisten. Bei Unklarheiten bezüglich der Zuteilung zu einer Sterbekategorie im Kreißsaal konnte mittels Diskussion eine Einigung zwischen Erstautorin und Letztautorin erreicht werden.

**Table:** **Tab. 1** Übersicht Mode of Death Kategorien Kreißsaal/NICU.

Mode of death Kreißsaal	
MOD KS 1	Totgeburt (Tod bei Ankunft im Krankenhaus)
MOD KS 2	Totgeburt (Bei Ankunft im Krankenhaus am Leben, Totgeburt bzw. Versterben in utero bei bewusster Entscheidung gegen eine operative Entbindung)
MOD KS 3	Totgeburt bei Schwangerschaftsabbruch aufgrund schwerer kongenitaler Fehlbildungen
MOD KS 4	Unterlassen einer Reanimation, Aufnahme NICU zur primär palliativen Betreuung bei extremer Frühgeburt, Fehlbildung und/oder infauster Prognose
MOD KS 5	Frustrane Reanimation
Mode of death NICU	
MOD NICU 1	Verstorben während kardiopulmonaler Reanimation
MOD NICU 2	Verstorben an der Beatmung bei Unterlassung weiterer Reanimationsmaßnahmen
MOD NICU 3	Verstorben bei Beendigung oder Vorenthalten lebenserhaltender Maßnahmen aufgrund von instabilen Vitalparametern
MOD NICU 4	Verstorben bei Beendigung lebenserhaltender Maßnahmen trotz stabiler Vitalparameter aus Gründen der Prognose und zu erwartenden Lebensqualität

Fußnote: Kategorie MOD KS 1–3 sind unterschiedliche Formen von Totgeburten. Die Kategorien MOD KS 4 und 5 beziehen sich auf Lebendgeborene.

Um Veränderungen über den Untersuchungszeitraum deutlicher herauszustellen, wurden die Daten zweier definierter Drei-Jahres-Zeitintervalle innerhalb von 10 Jahren (Zeitintervall I vom 01.01.2006–31.12.2008, Zeitintervall II vom 01.01.2013–31.12.2015) in gleicher Weise ausgewertet und verglichen.

## Statistische Auswertung

Die Datenerfassung und Datenanalyse erfolgte über Excel (Microsoft Inc. Redmond, WA, USA, Office 2019). Statistische Tests wurden außerdem über das Statistikprogramm „R“ durchgeführt. Für Gruppenvergleiche wurden bei normalverteilten Zielgrößen der t-Test, bei nicht-normalverteilten quantitativen Zielgrößen der „Wilcoxon-Mann-Whitney-Test“ und bei kategorialen Zielgrößen Fishers exakter Test angewendet. Das Signifikanzniveau wurde auf 0,05 gesetzt.

## Ergebnisse

**Abb. 1:**
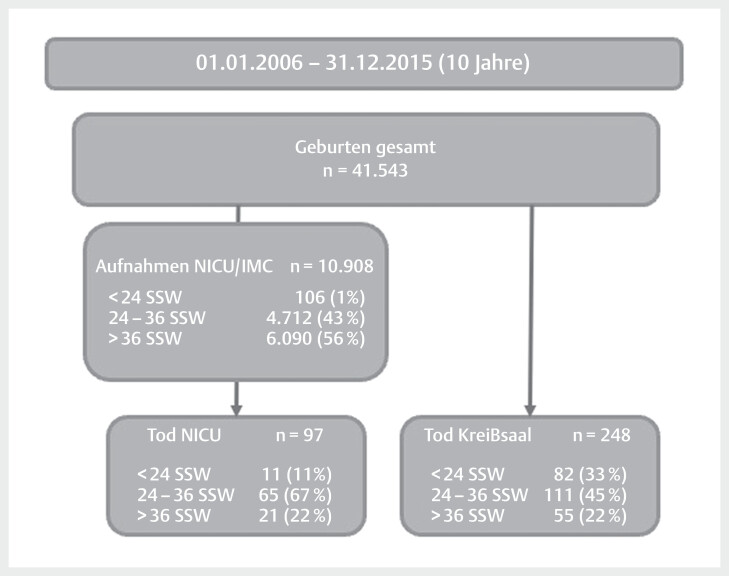
Übersicht Fallzahlen.

Während des 10-jährigen Beobachtungszeitraums wurden an der LMU München 41 543 Kinder entbunden. Gleichzeitig sind in dieser Zeitperiode 348 Kinder verstorben: 248 Kinder verstarben im Kreißsaal, 97 Kinder auf der NICU, und bei drei Kindern konnte anhand der Dokumentation nicht geklärt werden, wo sie verstorben sind, obwohl es naheliegt, dass diese am ehesten dem Kreißsaal zugeordnet werden können. Auf der Neugeborenen-Intensivstation (NICU) oder Überwachungsstation (IMC) wurden in 10 Jahren insgesamt 10 908 Kinder medizinisch betreut. Mit 56% waren mehr als die Hälfte der aufgenommenen Kinder Reifgeborene oder späte Frühgeborene (>35+0 SSW), und nur etwa 1% dieser Kinder waren Extremfrühgeborene an der Grenze zur Lebensfähigkeit (<24+0SSW). Auf der Neugeborenenintensivstation verstarben während des Untersuchungszeitraumes insgesamt 97 Neugeborene (Gesamtmortalität NICU 0,9%). ( Abb. 1 )

## Mode of Death

### Kreißsaal

Für 231 der 248 im Kreißsaal verstorbenen Neugeborenen konnte die Einteilung der MOD nach klinischen Kriterien vorgenommen werden ( Tab. 2 ). Insgesamt 59% der im Kreißsaal verstorbenen Kinder kamen bereits tot zur Welt. Von den 41% (n=95) Lebendgeborenen wurde die Mehrzahl der Kinder (n=83) nach einer gemeinsam getroffenen Entscheidung von Eltern und dem verantwortlichen Behandlungsteam primär palliativ im Kreißsaal betreut.

**Table:** **Tab. 2** Einteilung MOD Kreißsaal/NICU.

Mode of death Kreißsaal	gesamt 2006–2015 n=248*
MOD KS 1	Totgeburt (Tod bei Ankunft im Krankenhaus)	125 (54%)
MOD KS 2	Totgeburt (Bei Ankunft im Krankenhaus am Leben, Totgeburt bzw. Versterben in utero bei bewusster Entscheidung gegen eine operative Entbindung)	4 (2%)
MOD KS 3	Totgeburt bei Schwangerschaftsabbruch aufgrund schwerer kongenitaler Fehlbildungen	7 (3%)
MOD KS 4	Unterlassen einer Reanimation, Aufnahme NICU zur primär palliativen Betreuung bei extremer Frühgeburt, Fehlbildung und/oder infauster Prognose	83 (36%)
MOD KS 5	Frustrane Reanimation	12 (5%)
Mode of death NICU	gesamt 2006–2015 n=97**
MOD NICU 1	Verstorben während kardiopulmonaler Reanimation	3 (3%)
MOD NICU 2	Verstorben an der Beatmung bei Unterlassung weiterer Reanimationsmaßnahmen	8 (8%)
MOD NICU 3	Verstorben bei Beendigung oder Vorenthalten lebenserhaltender Maßnahmen aufgrund von instabilen Vitalparametern	43 (46%)
MOD NICU 4	Verstorben bei Beendigung lebenserhaltender Maßnahmen trotz stabiler Vitalparameter aus Gründen der Prognose und zu erwartenden Lebensqualität	40 (43%)

*Für die anderen Sterbefälle (n=17) konnten abgesehen vom Tod im Kreißsaal keinerlei weitere Dokumentation in den Aufzeichnungen gefunden werden und eine Zuteilung nicht erfolgen, auch wenn in diesen Fällen am ehesten von einer Totgeburt ausgegangen werden kann. **Im Fall von 3 Neugeborenen konnte die MOD Einteilung aufgrund fehlender Dokumentation nicht kategorisiert werden. Die Patientenakten enthielten nicht genügend Informationen über den Zeitraum vor dem Tod, um diese Todesfälle zu kategorisieren.

Ein weitaus kleinerer Anteil der Neugeborenen, nur 12 Kinder (5%), verstarben während des gesamten Beobachtungszeitraums im Kreißsaal aufgrund einer frustranen Reanimation. Dabei wurde primär ein kurativer Therapieansatz verfolgt, dieser verlief aber entweder frustran, oder das Therapieziel wurde während der proaktiven Erstversorgung aufgrund des klinischen Zustands und der dann erst evidenten aussichtslosen Prognose geändert.

### NICU

Für 94 der 97 auf der NICU verstorbenen Neugeborenen konnte die MOD Einteilung vorgenommen werden. ( Tab. 2 ). Insgesamt verstarben 89% und damit die überwiegende Mehrzahl der Kinder nach einer bewussten Entscheidung, lebensverlängernde Maßnahmen aktiv zu beenden. Davon verstarben 46% (n=43) bei Beendigung lebenserhaltender Maßnahmen aufgrund von instabilen Vitalparametern und 43% (n=40) durch Beendigung lebenserhaltender Maßnahmen trotz stabiler Vitalparameter aus Gründen der zu erwartenden Lebensqualität.

### Veränderungen im Zeitverlauf

#### Kreißsaal

Beim Vergleich der beiden jeweils dreijährigen Zeitintervalle zu Beginn und am Ende des Beobachtungszeitraume (I: 2006–2008 und II: 2013–2015) zeigte sich ein leichter Rückgang der lebend geborenen und im Kreißsaal verstorbenen Kinder (39% auf 24%, p=0,08; Tab. 3 ). Der Anteil der Extremfrühgeborenen an den im Kreißsaal verstorbenen Neugeborenen zeigte sich im Vergleich dazu unverändert (33% vs. 25%, p=0,46). Die Anzahl der aufgrund eines Schwangerschaftsabbruchs bei pränatal bekannten, schwerwiegenden Fehlbildungen und/oder Chromosomenaberrationen mit infauster Prognose tot geborenen Kinder nahm vom Zeitintervall I zu Zeitintervall II tendentiell von 1% auf 8% aller im Kreißsaal betreuten tot geborenen Kinder zu (p=0,12).

**Table:** **Tab. 3** Zeitverlauf Kreißsaal.

	Zeitintervall I *2006–2008*	Zeitintervall II *2013–2015*	p	Gesamt 10 Jahre *2006–2015*
Geburten	11 881	12 831		41 543
davon<24 SSW	51 (0,4%)	51 (0,4%)		188 (0,5%)
Tod im Kreißsaal	74	73	0,64	248
mean GA (Wochen)	30	30		29
weibliches Geschlecht	31 (42%)	41 (59%)	0,12	109 (48%)
<24 SSW	23 (33%)	18 (25%)	0,46	82 (33%)
24–36 SSW	30 (42%)	39 (55%)	0,18	111 (45%)
>36 SSW	18 (25%)	14 (20%)	0,56	55 (22%)
Lebendgeborene	28 (39%)	17 (24%)	0,08	95 (41%)
Lebensdauer (Minuten)*	52 (1–156)	33 (17–132)	0,99	52 (1–343)
Fehlbildungen/Chromo-somale Abberationen	20 (29%)	20 (29%)	1	63 (28%)
MOD KS 1	41 (58%)	48 (68%)	0,3	
MOD KS 2	1 (1%)	0 (0%)	1	
MOD KS 3	1 (1%)	6 (8%)	0,12	
MOD KS 4	26 (37%)	15 (21%)	0,06	
MOD KS 5	2 (3%)	2 (3%)	1	

GA: Schwangerschaftsdauer; SSW: Schwangerschaftswochen; MOD KS: mode of death Kreißsaal; MOD KS 1: Totgeburt (Tod bei Ankunft im Krankenhaus); MOD KS 2: Totgeburt (Bei Ankunft im Krankenhaus am Leben, Totgeburt bzw. Versterben in utero bei bewusster Entscheidung gegen eine operative Entbindung); MOD KS 3: Totgeburt bei Schwangerschaftsabbruch aufgrund schwerer kongenitaler Fehlbildungen und/oder infauste Prognose; MOD KS 4: Unterlassen einer Reanimation, Aufnahme NICU zur primär palliativen Betreuung bei extremer Frühgeburt, Fehlbildung und/oder infauster Prognose; MOD KS 5: Frustrane Reanimation; *Lebensdauer in Minuten; **für 3 Kindern in Zeitinterval I und 2 Kindern in Zeitinterval II konnte keine Einteilung vorgenommen werden

#### NICU

Auf der NICU wurden im Zeitintervall I insgesamt mehr Kinder behandelt als im Zeitintervall II, der Anteil der Extremfrühgeborenen stieg hingegen tendentiell von 28 auf 33 Kinder (0,8% vs 1,1%). In beiden Zeitintervallen kamen gleich viele Frühgeborene an der Grenze der Lebensfähigkeit zur Welt (n=51; Tab. 3 ). Die Mortalität im Kreissaal dieser Gruppe sank von 45% im Zeitintervall I (23 von 51 Kindern<24SSW) auf 35% im Zeitintervall II (18 von 51 Kindern<24SSW). Gleichzeitig wurden im Zeitintervall I weniger Extremfrühgeborene auf der NICU aufgenommen (28 Kinder im Zeitintervall I, 0,8% aller Aufnahmen, und 33 Kinder im Zeitintervall II, 1,1% aller Aufnahmen). Von den Extremfrühgeborenen sind im Zeitintervall I mit 6 von insgesamt 28 (21%) mehr Kinder auf der NICU verstorben als im Zeitintervall II mit 3 von insgesamt 33 (9%). In der Gruppe der Gesamtfrühgeborenen (24+0 bis 34+6 SSW) starben im Zeitintervall I deutlich mehr Frühgeborene als im Zeitintervall II. Der Anteil sank von 74% auf 50% (p=0,06, Tab. 4 ), bezogen auf alle Verstorbenen auf der NICU. Der Anteil der verstorbenen Reifgeborenen zeigte im Gegensatz von Zeitintervall I zu II eine Zunahme (9% auf 38%, p=0,01). Gleichzeitig war die Anzahl der Extremfrühgeborenen im Zeitintervall I niedriger, und deren Geburtsgewicht war in diesem Zeitraum deutlich höher. Dies erklärt auch den signifikanten Anstieg des medianen Geburtsgewichts der verstorbenen Kinder auf der NICU von 700 g auf 1.890 g (p=0,001). Von den Lebendgeborenen, die auf der NICU verstarben,wurde die Mehrzahl der Kinder primär palliativ betreut (MOD 4), von Zeitintervall I zu II verstarben weniger Kinder in diesem Kontext (37% vs. 21%); p=0,06). Während einer kardiopulmonalen Reanimation verstarben auf der NICU über den gesamten Zeitraum nur wenige Neugeborene und die Anzahl zeigte von Zeitintervall I zu II eine rückläufige Tendenz. An der Beatmung bei Unterlassung weiterer Reanimationsmaßnahmen sind in Zeitintervall I 15% der Kinder verstorben, in Zeitintervall II keines der Kinder Fälle (p=0,07). Im Gegensatz dazu gab es von Zeitintervall I zu II einen Anstieg der Kinder, für die nach einer bewussten Entscheidung, lebensverlängernde Maßnahmen beendet wurden (MOD 3+4). Im Zeitintervall I verstarben 79% der Neugeborenen nach einer solchen Entscheidung, in Zeitintervall II 96% . Insbesondere hinsichtlich des MOD 3 „Versterben bei Beendigung lebenserhaltender Maßnahmen aufgrund von instabilen Vitalparametern“ zeigt sich von Zeitintervall I zu II ein signifikanter Anstieg von 44 auf 72% (p=0,04).

**Table:** **Tab. 4** Zeitverlauf NICU.

	Zeitintervall I *2006–2008*	Zeitintervall II *2013–2015*	p	Gesamt 10 Jahre *2006–2015*
NICU Aufnahmen	3435	3094		10980
<24 SSW	28 (0,8%)	33 (1,1%)	0,35	106 (1%)
24–36 SSW	1511 (44%)	1324 (43%)	0,35	4712 (43%)
>36 SSW	1896 (55%)	1737 (56%)	0,46	6090 (56%)
Tod auf NICU	35 (1%)	26 (0,8%)	0,53	97 (0,9%)
Gewicht	700 (375–4400)	1890 (660–3950)	<0,001*	780 (375–4600)
Weibliches Geschlecht	11 (31%)	14 (54%)	0,13	41 (42%)
<24 SSW	6 (17%)	3 (12%)	0,72	11 (11%)
24–36 SSW	26 (74%)	13 (50%)	0,06	65 (67%)
>36 SSW	3 (9%)	10 (38%)	0,01*	21 (22%)
Lebenstage (median)	7 (1–41)	4 (1–42)	0,91	5 (1–106)
MOD NICU 1	2 (6%)	1 (4%)	1	
MOD NICU 2	5 (15%)	0 (0%)	0,07	
MOD NICU 3	15 (44%)	18 (72%)	0,04*	
MOD NICU 4	12 (35%)	6 (24%)	0,4	

SSW: Schwangerschaftswochen; Gewicht in Gramm; MOD NICU: mode of death NICU; MOD NICU 1: Versterben während kardiopulmonaler Reanimation; MOD NICU 2: Versterben an der Beatmung bei Unterlassung weiterer Reanimationsmaßnahmen; MOD NICU 3: Versterben bei Beendigung oder Vorenthalten lebenserhaltender Maßnahmen aufgrund von instabilen Vitalparametern; MOD NICU 4: Versterben bei Beendigung lebenserhaltender Maßnahmen trotz stabiler Vitalparameter aus Gründen der Prognose und zu erwartenden Lebensqualität; * signifikant p<0,05

## Diskussion

Die hier vorgelegten Daten zeigen, dass an unserem Zentrum schwer kranke Neugeborene sowohl im Kreißsaal als auch auf der Neugeborenenintensivstation in der überwiegenden Mehrzahl und zunehmend nach einer bewussten Entscheidung, lebensunterstützende Maßnahmen nicht durchzuführen oder diese aktiv zu beenden, verstarben.

Darüber hinaus verstarben in jüngerer Zeit mehr Kinder durch einen Schwangerschaftsabbruch bei pränatal diagnostizierten Fehlbildungen und infauster Prognose und weniger Kinder infolge frustranen Reanimationen. Es wurden in jüngerer Zeit mehr Frühgeborene an der Grenze der Lebensfähigkeit proaktiv versorgt, und weniger dieser Kinder verstarben postnatal.

Ein großer Anteil der im Kreißsaal verstorbenen Neugeborenen kam bereits tot auf die Welt und bestätigt damit Angaben aus der Literatur 21 22 . Totgeburten wurden bei bisherigen Datenerhebungen zur Untersuchung neonataler Todesfälle häufig ausgeschlossen 1 2 5 17 23 . Allerdings geht auch einer Totgeburt oftmals eine Entscheidung hinsichtlich des Therapieziels voraus. Auch wenn die meisten Kinder bereits bei Ankunft im Krankenhaus verstorben waren, belegen die ansteigenden Zahlen für die Totgeburten bei Schwangerschaftsabbruch aufgrund schwerer kongenitaler Fehlbildungen jüngerer Zeit (Zeitintervall II) eine bemerkenswerte Entwicklung. Der hier beobachtete Trend bestätigt Daten aus der internationalen Literatur und spiegelt auch für Deutschland die soziokulturelle Entwicklung im Wandel der Zeit wider 24 25 26 .

Die signifikante Zunahme des Anteils der verstorbenen Reifgeborenen in jüngerer Zeit lässt sich nur zum Teil durch ein verschobenes Verhältnis zugunsten des häufigeren Überlebens Frühgeborener erklären. Möglicherweise wurde darüber hinaus bei mehr Reifgeborenen mit schwerwiegenden Erkrankungen initial ein Therapieversuch unternommen.

Auf der NICU verstarben im Zeitintervall II nahezu alle Neugeborenen nach einer bewussten Entscheidung, lebenserhaltende Maßnahmen zu beenden.

Betrachtet man zusammengefasst den Wandel in der demographischen Entwicklung sowie in der Zuordnung zu den MOD Kategorien, so ergeben sich zwei bedeutsame Unterschiede im Hinblick auf das Behandlungsregime schwer kranker Früh- und Neugeborener: i) Todesfälle unter Reanimation und trotz lebenserhaltender Maßnahmen ereigneten sich zunehmend seltener. ii) Sowohl im Kreißsaal als auch auf der Neugeborenenintensivstation wurden mehr aktive Entscheidungen im Hinblick auf das Therapieziel getroffen. Die steigende Schwangerschaftsabbruchsrate wie auch die Dynamik innerhalb der Sterbekategorien sprechen möglicherweise für ein vermehrtes Bedürfnis von Eltern und medizinischem Personal, auf Grundlage des klinischen Zustands oder der zu erwartenden Lebensqualität diese schwerwiegenden Entscheidungen zu treffen und umzusetzen. Offenbar findet ein zunehmend kritischer Umgang hinsichtlich der Indikation und des Ausschöpfens intensivmedizinischer Maßnahmen statt. Gleichzeitig lässt sich durch ein progressiveres Vorgehen an der Grenze der Lebensfähigkeit eine erhöhte Bereitschaft erkennen, diesen unreifen Kindern mit einem hohen Risiko für schwere Komplikationen mit einem vorerst proaktiven Therapieansatz zumindest ein Entscheidungsfenster zu öffnen und eine Lebensperspektive zu bieten.

Die meisten Studien, welche eine Datenerhebung zu Therapiezieländerungen bei Neugeborenen durchführten, wählten Kriterien ähnlich der hier beschriebenen Untersuchung, um die Thematik greifbarer und eine Einteilung zum Vergleich der Patientengruppen möglich zu machen. Diese Kriterien stützten sich im Wesentlichen auf 3 Säulen: i) den physiologischen Zustand bzw. die Vitalparameter des Kindes, ii) Maßnahmen, die durchgeführt, nicht durchgeführt, oder beendet wurden, und iii) die Intention, mit welcher über (nicht-) durchgeführte Maßnahmen entschieden wurde, zum Beispiel ein nicht mehr abwendbarer Tod, unverhältnismäßiges Leiden oder ein hohes Risiko für eine eingeschränkte Prognose und Lebensqualität.

Verhagen et al. betonten bereits, dass unterschiedliche Ergebnisse zum Treffen von *End-of-life Decisions* allein durch eine unterschiedliche Interpretation dieser Kriterien zustande kommen können 15 . Beispielsweise wurde in der Studie von Verhagen et al. eine Beatmung, welche bei sterbenden Neugeborenen aktiv beendet wurde, als eine Entscheidung zur Therapiezieländerung angesehen. In anderen Untersuchungen wurden Fälle mit vergleichbaren Rahmenbedingungen als nicht klassifizierbar eingestuft oder als Tod trotz maximaler Ausschöpfung lebensverlängernder Maßnahmen 4 16 . Wenn allerdings ein einheitliches Kategorisierungsschema mit feststehenden Definitionen von „withholding/withdrawing life sustaining therapy“ in Korrelation mit dem klinischen Zustand des Neugeborenen bzw. stabiler oder instabiler Vitalparameter verwendet wird, ist ein Vergleich möglich 5 . Eine Gegenüberstellung der hier vorgestellten Ergebnisse mit bereits publizierten Daten muss deshalb aufgrund eines gewissen Interpretationsspielraumes der uneinheitlichen Kategorisierung als bedingt aussagekräftig eingestuft werden. Zudem berichten Studien, die sich mit diesem Thema befassten, häufiger über die Einstellung der behandelnden Mediziner, als über die tatsächlich durchgeführten Standards und Praktiken 25 26 27 28 29 30 . Selbst wenn über das Beenden durchgeführter medizinischer Maßnahmen berichtet wird, wird selten differenziert zwischen den Faktoren, die zur Entscheidungsfindung beitrugen 16 31 32 33 34 .

Ein Vergleich zwischen unterschiedlichen Perinatalzentren und/oder Ländern, sowie unterschiedlichen Studien und Datenerhebungen zu den praktizierten Therapiezieländerungsstrategien und Entscheidungsfindungsprozessen ist komplex – erscheint aber dennoch wichtig.

Das hier verwendete Kategorisierungsschema beruht auf der Untersuchung von Koper et al. und somit können die Daten der niederländischen Kohorte aus zwei ähnlichen Zeitintervallen für einen Vergleich herangezogen werden. Dieser Vergleich ist aus besonders interessant, da in den Niederlanden im Bezug auf eine mögliche Therapiezieländerungen zugunsten eines palliativen Therapieziels sowie Lebensbeendigung auf Verlangen und Hilfe zur Selbsttötung sowie Euthanasie eine liberalere Haltung im Vergleich zu Deutschland verbreitet ist. An den Perinatalzentren des LMU Klinikums München wurden in den beiden Zeitintervallen mehr als doppelt so viele Kinder geboren und stationär betreut, wie in der Untersuchung von Koper et al. Dafür verstarben in München im Vergleich zur niederländischen Kohorte weniger Kinder (1% vs 7% der NICU Aufnahmen). Relevante Unterschiede ergaben sich insbesondere hinsichtlich der Veränderungen über die Zeit und in der Versorgung Extremfrühgeborener an der Grenze der Lebensfähigkeit: Im Kreißsaal verstarben in der niederländischen Kohorte nahezu doppelt so viel Neugeborene wie in München. Der Anteil der im Kreißsaal verstorbene Extremfrühgeborenen im Graubereich der „Grenze der Lebensfähigkeit“ war in München von Zeitintervall I zu II leicht abnehmend, in der niederländische Kohorte war dieser Anteil von Zeitintervall I zu II deutlich angestiegen. Die Mortalität der Kinder an der Grenze der Lebensfähigkeit war in München geringer im Vergleich zur niederländischen Kohorte; 21% vs. 67% jeweils in Zeitintervall I und 9% vs. 100% in Zeitintervall II.

Trotz geographischer Nähe, sozioökonomischer Ähnlichkeit und vergleichbarer medizinischer Versorgungsqualität zwischen den Niederlanden und Deutschland zeigen sich hier bemerkenswerte Diskrepanzen. Eine mögliche Erklärung findet sich in kulturellen Unterschieden zwischen Deutschland und den Niederlanden. Eine große 10-Länder-übergreifende europäische Studie aus dem Jahr 2000 konnte belegen, dass sich die Grundhaltung des behandelnden Arztes in seinen ethischen Entscheidungen wiederfindet, und zum anderen, dass die jeweiligen Grundhaltungen maßgeblich durch die Länderzugehörigkeit beeinflusst werden 28 . Es wird hierbei für die Niederlande eine Haltung beschrieben, welche von allen eingeschlossenen Ländern den Fokus im Entscheidungsfindungsprozess am stärksten auf eine möglichst gute Lebensqualität legt („quality-of-life attitude“), währenddessen in Deutschland eine grundsätzlich lebensbejahende Einstellung („pro-life attitude“) einen höheren Stellenwert einnimmt.

Dass sich die Größen der Kohorten der LMU München und der Niederlande in den verglichenen 3-Jahres-Zeitintervallen deutlich unterschieden und mehr als doppelt so viele Kinder in der Münchener Kohorte auf die Welt kamen und ebenfalls mehr als doppelt so viele stationär betreut wurden, kann durch die geschilderten gesundheitspolitischen Systemunterschiede gut erklärt werden. Noch deutlicher wird dieser Unterschied, wenn man die verstorbenen Kinder auf der Neugeborenenintensivstation in Relation setzt zur Anzahl der insgesamt stationär behandelten Kinder. Vergleicht man die Mortalität, so verstarb in den Niederlanden ein um ein Vielfaches höherer Anteil der intensivmedizinisch betreuten Neugeborenen auf der NICU. Dies wird mitbegründet durch die Tatsache, dass in einem niederländisches Krankenhaus per se fast nur Geburten mit Risikokonstellation stattfinden oder kranke Neu- und Frühgeborene zuverlegt werden. Der Anstieg von im Kreißsaal verstorbenen Extremfrühgeborenen an der Grenze der Lebensfähigkeit von 22 auf 42% in den Niederlanden unterscheidet sich wesentlich zu den hier untersuchten Sterbefälle, die einen Rückgang von 33 auf 25% für diese Gruppe belegen. Die Veröffentlichung des Groningen-Protokolls zur Euthanasie bei Neugeborenen könnte diese Entwicklung erklären, da dadurch unter strengen Vorgaben eine Euthanasie eines schwer erkrankten Neugeborenen ermöglicht wurde 35 .

## Schlussfolgerung

Ein primär proaktiverer Ansatz bereits pränatal hat in Deutschland in den letzten Jahren dazu geführt, dass die primäre Palliativversorgung Extremfrühgeborener häufiger abgelöst wird durch einen Therapieversuch an der Grenze der Lebensfähigkeit. Dies steht im Gegensatz zu anderen Ländern, hier beispielhaft die Niederlande. Da keine Änderung der deutschen Leitlinie zum Vorgehen bei Frühgeburtlichkeit an der Grenze der Lebensfähigkeit während des Datenerhebungszeitraumes vorgenommen wurde, ist diese Veränderung eher dem Zusammenwirken elterlicher Wunsch- und Erwartungshaltung und ärztlichem Handeln zuzuschreiben.

Ein primär proaktiverer Ansatz vermittelt eine höhere Bereitschaft, unreifen oder kranken Kindern mit einem erhöhten Risiko für schwere Komplikationen mit einem vorerst proaktiven Therapieansatz zumindest ein Entscheidungsfenster zu öffnen und eine Lebensperspektive zu bieten.

Dieses Vorgehen setzt eine bewusste und klare Kommunikation mit Eltern voraus. Das Öffnen eines Entscheidungsfensters erfordert die Abwägung von einerseits elterlichen Präferenzen und Bedürfnissen und andererseits ärztlicher Einschätzung von Prognose. Wenn Ärzte in der Zukunft zunehmend diese Prozesse begleiten werden, sollte die Aneignung von benötigten Kommunikationsstrategien und Kenntnissen des Entscheidungsfindungsprozesses in der Weiterbildung an Bedeutung gewinnen. Darüber hinaus sollte versucht werden, für die Umsetzung der angewandten Methoden in der Betreuung kritisch kranker und sterbender Früh- und Neugeborener, Handlungsleitfäden zu etablieren, die Symptome unverhältnismäßigen Leidens sowie Aspekte von Prognose und Lebensqualität berücksichtigen.

## References

[1] Schulz-Baldes A Huseman D Loui A Neonatal end-of-life practice in a German perinatal centre Acta Paediatr 2007 96 681 687 10.1111/j.1651-2227.2007.00234.x 17462059

[2] Berger T M Hofer A Causes and circumstances of neonatal deaths in 108 consecutive cases over a 10-year period at the Children’s Hospital of Lucerne, Switzerland Neonatology 2009 95 157 163 10.1159/000153100 18776730

[3] Roy R Aladangady N Costeloe K Decision making and modes of death in a tertiary neonatal unit Arch Dis Child Fetal Neonatal Ed 2004 89 F527 F530 10.1136/adc.2003.032912 15499147 PMC1721774

[4] Barton L Hodgman J E The contribution of withholding or withdrawing care to newborn mortality Pediatrics 2005 116 1487 1491 10.1542/peds.2005-0392 16322175

[5] Verhagen A A Janvier A Leuthner S R Categorizing neonatal deaths: a cross-cultural study in the United States, Canada, and The Netherlands J Pediatr 2010 156 33 37 10.1016/j.jpeds.2009.07.019 19772968

[6] Verhagen A A van der Hoeven M A van Meerveld R C Physician medical decision-making at the end of life in newborns: insight into implementation at 2 Dutch centers Pediatrics 2007 120 e20 e28 10.1542/peds.2006-2555 17606544

[7] Wall S N Partridge J C Death in the intensive care nursery: physician practice of withdrawing and withholding life support Pediatrics 1997 99 64 70 10.1542/peds.99.1.64 8989340

[8] Weiner J Sharma J Lantos J How infants die in the neonatal intensive care unit: trends from 1999 through 2008 Arch Pediatr Adolesc Med 2011 165 630 634 10.1001/archpediatrics.2011.102 21727274

[9] Bundesamt DS. In; 2022

[10] Lawn J E Blencowe H Oza S Every Newborn: progress, priorities, and potential beyond survival Lancet 2014 384 189 205 10.1016/S0140-6736(14)60496-7 24853593

[11] Lehtonen L Gimeno A Parra-Llorca A Early neonatal death: A challenge worldwide Semin Fetal Neonatal Med 2017 22 153 160 10.1016/j.siny.2017.02.006 28238633

[12] Wilkinson D J Fitzsimons J J Dargaville P A Death in the neonatal intensive care unit: changing patterns of end of life care over two decades Arch Dis Child Fetal Neonatal Ed 2006 91 F268 F271 10.1136/adc.2005.074971 16790729 PMC2672727

[13] Morris J K Wald N J Quantifying the decline in the birth prevalence of neural tube defects in England and Wales J Med Screen 1999 6 182 185 10.1136/jms.6.4.182 10693061

[14] Duff R S Campbell A G Moral and ethical dilemmas in the special-care nursery N Engl J Med 1973 289 890 894 10.1056/NEJM197310252891705 4729120

[15] Verhagen A A Dorscheidt J H Engels B End-of-life decisions in Dutch neonatal intensive care units Arch Pediatr Adolesc Med 2009 163 895 901 10.1001/archpediatrics.2009.166 19805707

[16] Hagen C M Hansen T W Deaths in a neonatal intensive care unit: a 10-year perspective Pediatr Crit Care Med 2004 5 463 468 10.1097/01.pcc.0000128893.23327.c1 15329163

[17] Tudehope D Papadimos E Gibbons K Twelve-year review of neonatal deaths in the delivery room in a perinatal tertiary centre J Paediatr Child Health 2013 49 E40 E45 10.1111/jpc.12021 23198828

[18] Doyle L W Anderson P J Battin M Long term follow up of high risk children: who, why and how? BMC Pediatr 2014 14 279 10.1186/1471-2431-14-279 25399544 PMC4289257

[19] Berger T M Decisions in the gray zone: evidence-based or culture-based? J Pediatr 2010 156 7 9 10.1016/j.jpeds.2009.08.044 20006758

[20] World Medical A. World Medical Association Declaration of Helsinki: Ethical Principles for Medical Research Involving Human Participants JAMA 2024 10.1001/jama.2024.21972.doi:10.1001/jama.2024.21972 39425955

[21] Koper J F Bos A F Janvier A Dutch neonatologists have adopted a more interventionist approach to neonatal care Acta Paediatr 2015 104 888 893 10.1111/apa.13050 26014464

[22] Berger T M Steurer M A Bucher H U Retrospective cohort study of all deaths among infants born between 22 and 27 completed weeks of gestation in Switzerland over a 3-year period BMJ Open 2017 7 e015179 10.1136/bmjopen-2016-015179 PMC5734457 28619775

[23] Garten L von der Hude K Palliative Care in the Delivery Room: Challenges and Recommendations Children (Basel) 2022 10 10.3390/children10010015 PMC9856529 36670565

[24] Cuttini M Nadai M Kaminski M End-of-life decisions in neonatal intensive care: physicians’ self-reported practices in seven European countries EURONIC Study Group. Lancet 2000 355 2112 2118 10.1016/s0140-6736(00)02378-3 10902625

[25] Barr P Relationship of neonatologists’ end-of-life decisions to their personal fear of death Arch Dis Child Fetal Neonatal Ed 2007 92 F104 F107 10.1136/adc.2006.094151 17284476 PMC2675451

[26] Norup M Limits of neonatal treatment – a survey of attitudes in the Danish population Journal of Medical Ethics 1998 24 200 206 10.1136/jme.24.3.200 9650116 PMC1377524

[27] Singh J Fanaroff J Andrews B Resuscitation in the “gray zone” of viability: determining physician preferences and predicting infant outcomes Pediatrics 2007 120 519 526 10.1542/peds.2006-2966 17766524

[28] Rebagliato M Cuttini M Broggin L Neonatal end-of-life decision making: Physicians’ attitudes and relationship with self-reported practices in 10 European countries JAMA 2000 284 2451 2459 10.1001/jama.284.19.2451 11074774

[29] Saigal S Stoskopf B Feeny D Differences in Preferences for Neonatal Outcomes Among Health Care Professionals, Parents, and Adolescents JAMA 1999 281 1991 1997 10359387 10.1001/jama.281.21.1991

[30] Streiner D Saigal S Burrows E Attitudes of Parents and Health Care Professionals Toward Active Treatment of Extremely Premature Infants Pediatrics 2001 108 152 157 11433068 10.1542/peds.108.1.152

[31] de Leeuw R Foregoing intensive care treatment in newborn infants with extremely poor prognoses J Pediatr 1996 129 661 666 8917230 10.1016/s0022-3476(96)70146-4

[32] Cuttini M Kaminski M Saracci R The EURONIC Project – a European concerted action on information to parents and ethical decision-making in neonatal intensive care Paediatr Perinat Epidemiol 1997 11 661 666 10.1046/j.1365-3016.1997.d01-29.x 9373868

[33] Cook L A Watchko J F Decision making for the critically ill neonate near the end of life J Perinatol 1996 16 133 136 8732563

[34] van der Heide A van der Maas P Medical end-of-life decisions made for neonates and infants in the Netherlands 1997 350 251 255 10.1016/S0140-6736(97)02315-5 9242802

[35] Verhagen E Sauer P J The Groningen protocol--euthanasia in severely ill newborns N Engl J Med 2005 352 959 962 10.1056/NEJMp058026 15758003

